# Miltefosine analogues with comparable antileishmanial activity and significantly reduced macrophage cytotoxicity

**DOI:** 10.1590/0074-02760240219

**Published:** 2025-05-26

**Authors:** Lais Alonso, Laís Flávia Nunes Lemes, George E Magoulas, Brenda de Lucena Costa, Rodrigo Saar Gomes, Miriam Leandro Dorta, Maria Laura Bolognesi, Luiz Antonio Soares Romeiro, Theodora Calogeropoulou, Antonio Alonso

**Affiliations:** 1 Universidade Federal de Goiás, Instituto de Física, Goiânia, GO, Brasil; 2 Universidade de Brasília, Faculdade de Medicina, Programa de Pós-Graduação em Medicina Tropical, Laboratório de Desenvolvimento de Inovações Terapêuticas, Brasília, DF, Brasil; 3 Universidade Católica de Brasília, Brasília, DF, Brasil; 4 National Hellenic Research Foundation, Institute of Chemical Biology, Athens, Greece; 5 Universidade Federal de Goiás, Instituto de Patologia Tropical e Saúde Pública, Departamento de Imunologia e Patologia Geral, Goiânia, GO, Brasil; 6 University of Bologna, Department of Pharmacy and Biotechnology, Bologna, Italy; 7 Universidade de Brasília, Faculdade de Ciências da Saúde, Departamento de Farmácia, Brasília, DF, Brasil

**Keywords:** miltefosine analogues, Leishmania, macrophage, erythrocyte, electron paramagnetic resonance

## Abstract

**BACKGROUND:**

Miltefosine (MIL) is the only oral drug approved for leishmaniasis treatment, but its use is limited by gastrointestinal toxicity. Novel alkylphospholipid analogues may provide safer and more effective alternatives.

**OBJECTIVES:**

This study aimed to assess the antileishmanial activity, cytotoxicity, and membrane interactions of three MIL analogues TC387, TC388, and TC437 against *Leishmania amazonensis.*

**METHODS:**

Antileishmanial and cytotoxic activities were evaluated in *L. amazonensis*, J774.A1 macrophages, and erythrocytes. Membrane interactions were characterized using spin-label electron paramagnetic resonance (EPR) spectroscopy.

**FINDINGS:**

TC387, TC388, and TC437 demonstrated EC_50_ values of 10-16 µM for intracellular amastigotes, compared to 17 µM for MIL, with selectivity indices (SI) ranging from 43-163, significantly higher than MIL’s SI of 5. EPR data revealed that the analogues increased membrane protein dynamics and caused greater disruption at the lipid-protein interface of parasite membranes relative to MIL. This disruption likely enhances pore formation, ion leakage, and reactive oxygen species (ROS) production, leading to parasite death.

**MAIN CONCLUSIONS:**

The MIL analogues TC387, TC388, and TC437 exhibited superior SI and comparable or slightly enhanced antileishmanial activity relative to MIL, along with very low hemolytic potential. These findings support further investigation of these analogues as promising oral therapeutic candidates for leishmaniasis.

Leishmaniasis is a neglected tropical disease caused by over 20 species of *Leishmania*, affecting more than 90 countries, and placing approximately 350 million people at risk. Annually, an estimated 1.5 to 2 million new cases occur.^1^ ^,^ ^2^ *Leishmania amazonensis* is associated with cutaneous leishmaniasis, particularly the diffuse form, a rare manifestation where parasites proliferate uncontrollably, resulting in widespread, non-ulcerative skin lesions.^1^ Current treatments include pentavalent antimonials, amphotericin B, miltefosine (MIL), and paromomycin.^3^ However, the limited availability of new antileishmanial agents coupled with toxicity, high costs, and increasing drug resistance, underscores the urgent need for novel therapeutic options.^3^

MIL is a synthetic phospholipid analogue (hexadecylphosphocholine) that is used in the Indian subcontinent as a first-line treatment for post-kala-azar dermal leishmaniasis (PKDL).^4^ In Brazil, the Ministry of Health decided to incorporate MIL as a first-line treatment for cutaneous leishmaniasis within Brazil’s Unified Health System (the Sistema Único de Saúde - SUS) (DOU, Portaria Nº 56, October 30, 2018). MIL has a broad spectrum of activity, including activities against schistosomiasis mansoni,^5^ *Giardia lamblia* ^6^ and particularly against several *Leishmania* species.^7^ ^,^ ^8^ However, MIL is teratogenic and as an oral drug, has several side effects related to its zwitterionic surfactant properties, which include gastrointestinal discomfort, anorexia, nausea, vomiting, and diarrhoea.^9^ In addition, the mechanisms of action for MIL are still not well established.

Using electron paramagnetic resonance (EPR) spectroscopy associated with the spin label method, our group has previously shown that MIL causes substantial increases in the dynamics of *L. amazonensis* plasma membrane proteins for concentrations of the drug in the range of leishmanicidal activity.^10^ ^,^ ^11^ ^,^ ^12^ Although several mechanisms of action for MIL have been proposed,^7^ it is important to consider that a series of MIL-induced cellular changes reported in the literature could result from a primary attack on the plasma membrane. For instance, increased fluidity in the plasma membrane, particularly at the lipid-protein interface, can lead to electrolyte leakage and ionic imbalances, potentially altering mitochondrial membrane potential.^13^ Additionally, MIL has been shown to elevate intracellular Ca^2+^ levels.^14^ The association between changes in mitochondrial membrane potential and increased formation of reactive oxygen species (ROS) is well documented.^15^ ^,^ ^16^ In turn, increased ROS formation has been associated with plasma membrane rigidity^17^ ^,^ ^18^ and the triggering of apoptosis-like cell death in the *Leishmania* parasite.^19^

This work presents three MIL analogues, TC387, TC388, and TC437, with *in vitro* antileishmanial activity superior to that of MIL and significantly lower cytotoxicity, as evidenced by their higher selectivity indices (SI) for *Leishmania* parasites. These new alkylphosphocholine derivatives were synthesised from cashew nutshell liquid (CNSL), following the waste-to-pharma concept.^20^ Unlike other reported MIL analogues, which typically modify the polar region, these compounds incorporate CNSL phenolic constituents into the lipid portion of MIL (Fig. 1). Spin label EPR spectroscopy was used to compare the effects of MIL and its analogues on the molecular dynamics of *L. amazonensis* promastigote. The EPR data, along with the biophysical parameters calculated, suggest that modifications to the MIL alkyl chain influence the compounds’ interactions at the lipid-protein interface, modulating both membrane dynamics and antiproliferative activity.

**Fig. 1:**
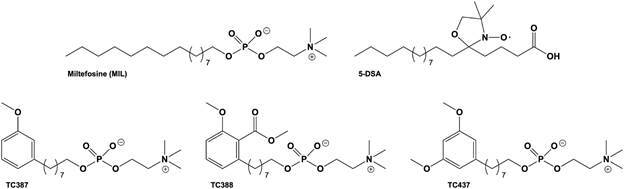
chemical structures of miltefosine (MIL), the MIL analogues, TC387, TC388, and TC437, and the spin label 5-DSA used in this study.

## MATERIALS AND METHODS

Chemicals - MIL was obtained from Avanti Polar Lipids Inc. (Alabaster, AL, USA). The following reagents were purchased from Sigma-Aldrich (St. Louis, MO, USA): 5-doxyl stearic acid (5-DSA), Grace’s insect medium, RPMI-1640 medium, L-glutamine, penicillin, streptomycin, hygromycin B, 3-(4,5-dimethylthiazol-2-yl)-2,5-diphenyltetrazolium bromide (MTT), and sodium bicarbonate. Heat-inactivated foetal calf serum (FCS) (Corning Life Sciences, Corning, NY, USA). The TC compounds were synthesised according to the method described by Nunes et al.^20^

Cells - *Leishmania (L.) amazonensis* (MHOM/BR/75/Josefa) and green fluorescent protein (GFP)-labelled *L. amazonensis* (IFLA/BR/67/PH8) reference strains in their promastigote form were grown in 24-well microtiter plates containing 2 mL of Grace’s insect medium supplemented with 20% heat-inactivated FCS, 2 mM L-glutamine, 100 U/mL penicillin, and 100 µg/mL streptomycin, as previously described.^11^ ^,^ ^12^ The promastigotes were collected for the experiments when the highest proportion of infective metacyclics was reached (6th day of culture). The J774.A1 murine macrophage cell line was obtained from the cell bank of Rio de Janeiro (NCE/UFRJ). Macrophages were cultured in RPMI-1640 medium supplemented with 10% FCS, 2 mM L-glutamine, 100 U/mL penicillin, and 100 μg/mL streptomycin at 36.5ºC in a humidified incubator containing 5% CO_2_.

*In vitro assays of antileishmanial activity and macrophage cytotoxicity* - Parasites at different cell concentrations (5 x 10^6^, 1 x 10^7^, 1 x 10^8^, and 1 x 10^9^ cells/mL), or J774.A1 macrophages at 1 x 10^6^ cells/mL, were treated with increasing concentrations of MIL or its analogues, TC387, TC388, and TC437 (Fig. 1), which were initially diluted in an ethanol/DMSO mixture (1:1, v/v) at 25 or 250 mg/mL and then diluted in culture medium supplemented with 10% FCS. Promastigote or macrophage samples (100 µL) containing six different concentrations of the compounds (5-160 μM for promastigotes and 200-6,400 μM for macrophages) were prepared in 96-well microtiter plates and incubated for 24 h at 26ºC for promastigotes or 36.5ºC for macrophages. Cell viability was quantified by measuring the reduction of MTT to formazan by mitochondrial reductases. Formazan absorbance was read on the enzyme-linked immunosorbent assay (ELISA) reader at 550 nm, and the half maximal effective concentration (EC_50_) or half-maximal cytotoxic concentration (CC_50_) values were determined based on the best fit of the sigmoidal curve on the absorbance data versus the compound concentrations.

*J774.A1 macrophage infection* - GFP-labelled *L. amazonensis* was cultivated in Grace’s insect medium supplemented as described above. The GFP-labelled parasites were selected using 30 μg/mL hygromycin B.^21^ J774.A1 macrophages were cultured in RPMI-1640 medium supplemented with 10% FCS, 2 mM L-glutamine, 11 mM sodium bicarbonate, 100 U/mL penicillin, and 100 µg/mL streptomycin. A sample with 4 x 10^6^ cells/mL was infected with GFP-*L. amazonensis* (five parasites/cell) from six-day cultures for 24 h. Following infection, the cells were washed with 1x phosphate-buffered saline (PBS) to remove non-internalised parasites and cultured for an additional 24 h in the presence of MIL or its analogues at different concentrations (7.5-240 μM).

After cooling to ~4ºC, samples were collected by mechanical harvesting of adherent cells, washed twice with 1x PBS, and fixed with 1% paraformaldehyde for flow cytometry analysis using a BD Accuri™ C6 Flow Cytometry instrument (BD Bioscience, San Jose, CA, USA). Macrophages were selected by forward versus side scatter (FSC *vs* SSC). The data were analysed using FCS Express software (De Novo Software^TM^, Glendale, CA, USA). The percentage of cells expressing GFP^+^ (% infected cells) and mean fluorescence intensity (estimated amount of internalised parasites) were evaluated. The mean EC_50_ values were determined based on the best-fit sigmoidal curve plotted for infection percentage versus compound concentration, considering the maximum and minimum percentages of infected cells.

*Haemolytic potential in whole blood and PBS* - Blood was collected from a university blood bank, adhering to an approved protocol by the Ethics Committee for Human and Animal Medical Research at Hospital das Clínicas, Universidade Federal de Goiás (CAAE: 81316417.1.0000.5078). To evaluate the haemolytic potential of MIL and its analogues (TC387, TC388, and TC437) in whole blood, plasma was separated by centrifugation at 1800 × g for 10 min at 4ºC. Next, 58 µL of plasma samples containing different concentrations of the compounds were prepared (in this case the compounds were diluted directly in blood plasma to final concentrations in the range of 2.5-12.5 mM), and 42 µL of the blood cells (100%) were added to each sample to reconstitute the whole blood. Samples were incubated for 24 h at 7 ± 1ºC, and several agitations were made in this period. After incubation, 1.4 mL PBS was added to each sample, and the tubes were centrifuged (3500 x g for 10 min). The percentage of haemolysis was determined based on the absorbance of haemoglobin in the supernatant at 540 nm relative to the sample without treatment (control). The compound concentration required for 50% haemolysis (HC_50_) was calculated by fitting the percentage of haemolysis versus compound concentration data to a sigmoidal curve.

To assess the haemolytic potential in PBS, blood was initially diluted threefold in PBS and centrifuged at 800 × g for 10 min at 4ºC. Plasma and white blood cells were removed, and the remaining cells were resuspended in PBS. Erythrocytes were washed twice in PBS, discarding the supernatant after centrifugation. Erythrocytes were then added to a micellar suspension of the compounds at various concentrations in PBS (0.3-10 mM), achieving a final haematocrit of 2%. Samples were incubated for 2 h at 36.5 ± 1ºC, and haemolysis percentages and HC_50_ values were determined as described above.

*EPR spectroscopy* - Promastigotes of *L. amazonensis* were cultured in medium without FCS at a concentration of 1.5 × 10^7^ parasites/mL in a total volume of 2 mL. The parasites were treated with MIL and its analogues, which were previously diluted in ethanol to a concentration of 50 mg/mL. The concentrations employed for treatment were 5, 10, 15, and 25 µM. Following 1-hour incubation at 26ºC, the samples were centrifuged at 25,000 × g for 10 min to remove the culture medium; under these conditions, any potential cell membrane fragments were also precipitated. The supernatant was discarded, and the parasites were washed once more with 1 mL of PBS before being resuspended in 50 µL of PBS. Each sample, containing 3 × 10^7^ parasites, was then spin-labelled with 5-DSA. To incorporate the spin label into the parasite membranes, a 0.5 µL aliquot of a 5-DSA ethanolic solution (5 mg/mL) was added to each 50 µL sample. For EPR measurements, the sample was transferred to a 1-mm inner diameter capillary tube, which was flame-sealed at one end. The capillary was subsequently centrifuged at 25,000 × g for 5 min, ensuring that the resulting parasite pellet, approximately 2 mm in height, was centred within the resonance cavity.

EPR measurements were recorded using the Bruker EMX-Plus spectrometer (Rheinstetten, Germany). EPR spectrometer settings were: modulation frequency, 100 kHz; modulation amplitude, 1.0 G; microwave power, 10 mW; magnetic field scan, 100 G; and sample temperature, 26ºC. The EPR spectra were simulated using the nonlinear least-squares (NLLS) software developed by Freed JH and co-workers.^22^ One of the main parameters of the NLLS after the best-fit process is the Rbar, the rate of rotational Brownian diffusion of the spin probe. As in other studies,^18^ Rbar was converted to the rotational correlation time, τ_c_, using the following equation:

$$\tau_{c}=\;1/6Rbar\;(1)$$

This study generated the best-fit spectra using a two-component spectral model, i.e., considering two populations of spin labels with different mobility states. In all simulations the principal values of the g- and A-tensors used for components 1 and 2 were as follows: g_xx_ (1) = g_xx_ (2) = 2.0078, g_yy_ (1) = g_yy_ (2) = 2.0058, g_zz_ (1) = g_zz_ (2) = 2.0028, A_xx_ (1) = 6.6 G, A_yy_ (1) = 7.0 G, A_zz_ (1) = 31.5 G, A_xx_ (2) = 5.5 G, A_yy_ (2) = 5.5 G, and A_zz_ (2) = 31.0 G. The NLLS program allows least squares fitting of the two-component experimental spectra, producing the motion parameters and from the less (1) and more (2) mobile components, as well as their relative fractions f_1_ and f_2_ in the spectrum. These parameters allowed us to calculate the average value of the motion parameter using the following equation:^22^ ^,^ ^23^

$$\tau_{c}=f_{1}\;\tau_{c1}+\;f_{2}\tau_{c2}\;(2)$$

*Statistical analysis* - Data expressed as means ± standard deviation (SD) were from at least three independent experiments. Comparisons between different groups were performed using one-way analysis of variance (ANOVA) and Tukey’s test to identify significant differences between the treatments for p < 0.05.

## RESULTS

**Fig. 2:**
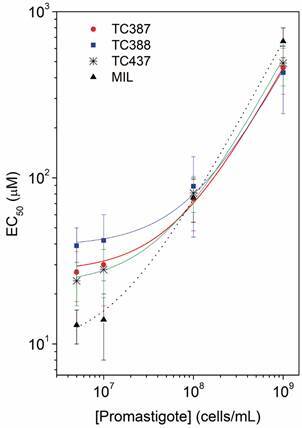
EC_50_ values of the compounds TC387, TC388, TC437, and miltefosine (MIL) for different cell (*Leishmania amazonensis* promastigotes) concentrations used in the experiment. The best-fit curves are presented.

*In vitro promastigote susceptibility assays for different cell concentrations* - To examine the interaction of MIL with the parasite membrane, as in previous studies,^10^ ^,^ ^12^ the dependence between the EC_50_ values of the compounds and the concentration of *L. amazonensis* parasite used in the experiment was evaluated. Fig. 2 shows the cell concentration-dependent behaviour for the EC_50_ values of the CNSL derivatives, TC387, TC388, and TC437, measured in parallel with MIL, for a more accurate comparison. For low cell concentrations, TC387, TC388, and TC437 had EC_50_ values higher than that of MIL, but for higher cell concentrations, such as in the range of 1 x 10^8^ to 1 x 10^9^ parasites/mL, the antiproliferative activities of MIL and its analogues were similar. As shown in Fig. 2, the increase in cell concentration from 1 x 10^7^ to 1 x 10^9^ parasites/mL (100x) led to increases in the EC_50_ values of MIL from ~13 to ~660 µM.

In cell suspension assays, the concentration of lipophilic compounds within the membrane can exceed that in the aqueous medium by over 10,000-fold (Log K_M/W_ > 4). In a well-diluted cell suspension, the quantity of compound associated with the membrane can be negligible due to the relatively small membrane volume; consequently, the compound predominantly resides in the aqueous phase, rendering its EC_50_ value effectively equivalent to its concentration in that phase, denoted as c_w50_. However, as cell concentration increases, the enhanced membrane volume results in a significant elevation of the EC_50_ compared to c_w50_. The equation that describes the variation of MIL EC_50_ values in relation to cell concentration in the assay has been derived and is expressed as follows:^10^ ^,^ ^11^

$${EC}_{50}=[(V_{mc}.\;c_{c}{)}^{-1}+\;K_{\frac{M}{W}}/\;(V_{mc}\;.\;c_{c}{)}^{-1}+1)]\;c_{w50}\;(3)$$

Where V_mc_ is the estimated cell membrane volume for an *L. amazonensis* promastigote, previously estimated as 8.17 x 10^-13^ mL,^11^ and c_c_ is the number of cells per mL. The parameters K_M/W_ and c_w50_ are covariant in eq. 3, and their values can be determined as best-fit parameters of eq. 3 for the EC_50_ against c_c_ data, shown in Fig. 2. For very dilute samples, the plasma membrane content is small, and the EC_50_ and c_w50_ values are practically the same; assuming in eq. 3 that (V_mc_ . c_c_)^-1^ >> K_M/W_, the EC_50_ = ~c_w50_. For high concentrations of cells, a remarkable amount of the compound goes to the membrane, and the EC_50_ value is much higher than the c_w50_ value. The c_m50_ value can also be determined from the K_M/W_ = c_w50_/c_m50_ relationship.

*Best-fit parameters obtained using eq. 3* - The parameters K_M/W_, c_w50,_ and c_w50_ obtained from the fitting of the curves shown in Fig. 2 are presented in Table I. MIL affinity for the parasite membrane was higher than for the CNSL derivatives, as indicated by the K_M/W_ values. This higher affinity for the promastigote membrane confers an advantage to MIL over its analogues at low cell concentrations; however, this advantage diminishes at elevated cell concentrations (Fig. 2). Although compound TC388 exhibited a lower affinity for the promastigote membrane and a higher c_w50_ value relative to compounds TC387 and TC437, the c_m50_ values of the three CNSL-alkylphospholipids were not significantly different (Table I). This observation suggests that the three MIL analogues possess comparable *in vitro* antileishmanial activities at higher cell concentrations.

**TABLE I:** Biophysical parameters associated with the interactions of miltefosine (MIL) and its analogues with plasma membranes of *Leishmania amazonensis* promastigotes

Compound	K_M/W_ (10^3^)^*a*^	log K_M/W_	c_w50_ (µM)	c_m50_ (M)
TC387	19.5 ± 3.7 (A)^*b*^	4.29	27.2 ± 2.9 (A)	0.53 ± 0.09 (A)
TC388	12.8 ± 2.8 (B)	4.11	38.9 ± 3.8 (B)	0.50 ± 0.13 (A)
TC437	24.7 ± 3.6 (A)	4.43	22.8 ± 3.7 (A)	0.56 ± 0.11 (A)
MIL	86.1 ± 5.5 (C)	4.94	9.4 ± 1.2 (C)	0.81 ± 0.07 (B)

*a*: best-fit parameters obtained by using eq. 3 on the data presented in Fig. 2; K_M/W_, membrane-water partition coefficient; cw_50_ and c_m50_, molecular concentrations in the aqueous phase and membrane, respectively, that inhibit parasite growth by half. *b*: in each column, measures indicated by the same capital letter are not statistically different with p < 0.05.

*Haemolytic potential and cytotoxicity in J774.A1 macrophages* - Table II shows the percentage of haemolysis in whole blood for compounds TC387, TC388, and TC437 compared to MIL. For this experiment, the test compound was diluted in blood plasma, and the blood was reconstituted with blood cells. A previous study reported an average of 53.2% haemolysis after treating blood with 2.5 mM MIL at 7ºC for 24 h.^24^ In the experiments conducted in this study under the same conditions, blood treatment with compounds TC387 and TC437 at three times the concentration resulted in 20.5% and 40.6% haemolysis, respectively, while 24.2% haemolysis was observed for 12.5 mM TC388.

**TABLE II:** Percentage of haemolysis in whole blood and PBS for miltefosine (MIL) and its analogues, TC387, TC388, and TC437

Compound (mM)	Whole blood - % haemolysis			
	MIL	TC387	TC388	TC437
2.5	53.2 ± 7.8^*a*^	--	--	--
7.5	--	22.5 ± 3.7	--	40.6 ± 7.4
10.0	--	75.3	14.5	92.5
12.5	--	--	24.2 ± 6.4	--
	Erythrocytes in PBS - % haemolysis			
0.3	54.0^*b*^	--	--	--
3.3	--	26.2	8.5	29.8
6.6	--	34.1	14.8	37.4
10.0	--	60.4	25.3	65.4

*a, b*: these data are from references^24^ and,^25^ respectively. PBS: phosphate-buffered saline.

In PBS solution, MIL analogs exhibited significantly lower haemolytic potential. For 300 µM MIL, approximately 54% haemolysis was previously reported at 20% haematocrit;^25^ in this study, compounds TC387, TC388, and TC437 showed less than 50% haemolysis, even at 22 times higher concentrations.

**Fig. 3:**
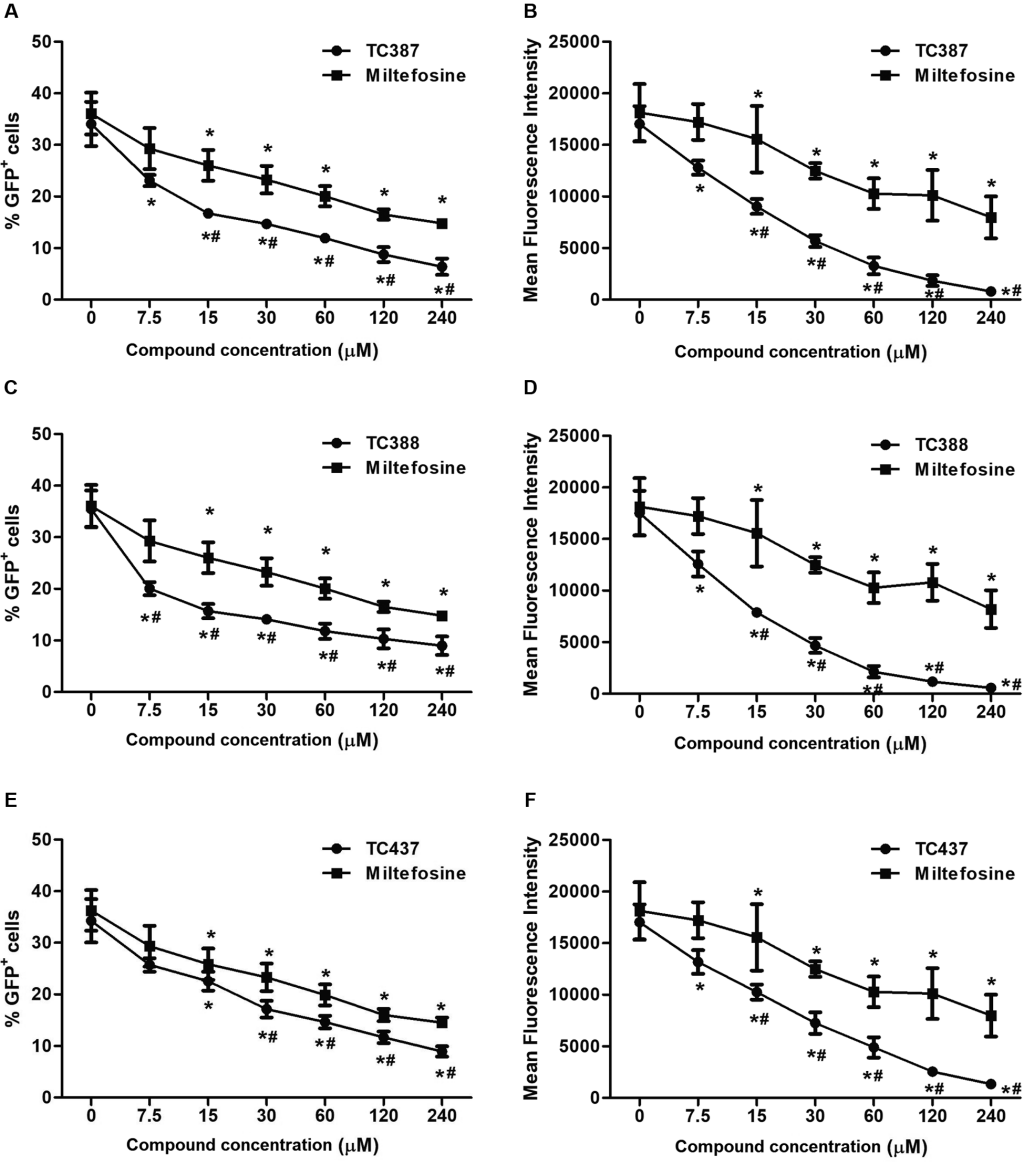
antileishmanial activity of compounds TC387, TC388, and TC437, and miltefosine (MIL) on *Leishmania amazonensis*-infected J774.A1 murine macrophages. % of GFP^+^ cells (infected macrophages) (panels A, C and E) and mean fluorescence intensity (MFI, estimated amount of internalised parasites) (panels B, E and F) were assessed by flow cytometry. ^*^ indicates a significant difference between the means for exposed and unexposed cells to compounds, and ^*#*^ indicates a significant difference between the means for cell treatments with MIL and its analogues (p < 0.05).

*Effect of MIL analogues against intracellular amastigotes* - To evaluate whether the CNSL derivatives TC387, TC388, and TC437 could eliminate *L. amazonensis* amastigotes within J774.A1 macrophages, we infected the macrophages with a GFP-expressing *L. amazonensis* strain, allowing quantification of infection rates using flow cytometry. The minimum concentrations of the compounds required to significantly reduce the percentage of macrophages infected with GFP-expressing amastigotes (Fig. 3, panels A, C and E) or to decrease the mean fluorescence intensity (Fig. 3, panels B, D and F) after 24 h of treatment were 15 µM for MIL and 7.5 µM for the three MIL analogs, except for TC437, where a significant reduction in the percentage of GFP+ cells compared to the untreated control was only observed at 15 µM. At a concentration of 30 µM, all MIL analogs demonstrated greater efficacy than MIL in reducing macrophage infection.

Interestingly, *L. amazonensis* amastigotes within macrophages were more susceptible to MIL analogs than promastigotes. While 7.5-15 µM of the MIL analogs caused a marked reduction in the amastigote population (Fig. 3), the C_w50_ values (equivalent to EC_50_ for diluted samples) for TC387, TC388, and TC437 were 27.2, 38.9, and 22.8 µM, respectively (Table I).

Table III summarises the antiproliferative and cytotoxic activity data for the compounds studied. The selectivity indices (SI) were significantly higher for the MIL analogues, particularly in assays involving amastigotes internalised within macrophages.

**TABLE III:** Antiproliferative activity against the protozoan parasite *Leishmania amazonensis* and cytotoxicity in J774A.1 cells

Samples	J774A.1	*L. amazonensis*			
		Promastigotes		Amastigotes	
	CC_50_ **±** SD (µM)	EC_50_ **±** SD (µM)	SI	EC_50_ **±** SD (µM)	SI
TC387	956 ± 277 (A) *	27 ± 9 (A)	34	12 ± 3 (AB)	80
TC388	1633 ± 404 (B)	39 ± 11 (A)	42	10 ± 2 (A)	163
TC437	692 ± 212 (C)	24 ± 7 (A)	29	16 ± 3 (BC)	43
Miltefosine	77 ± 23 (D)	13 ± 3 (B)	6	17 ± 3 (C)	5

CC_50_: cytotoxic concentration for 50% of the cells; EC_50_: half maximal effective concentration; SD: standard deviation; SI: selectivity index (CC_50_/EC_50_). ^*^Statistical significance: in each column, the means that do not have a capital letter in common are statistically different with p < 0.05

*MIL and its analogues increase the molecular dynamics in Leishmania membranes* - The spin label 5-DSA behaves as annular or boundary lipids in the cell membrane, preferentially surrounding the hydrophobic surface of membrane proteins.^26^ Owing to these interactions with the transmembrane proteins, 5-DSA can be used to monitor the molecular dynamics at the periphery of proteins in the lipid bilayer. Consequently, any changes in the 5-DSA spectra induced by MIL and its analogs are primarily attributed to alterations in the dynamics of the membrane protein component.

**Fig. 4:**
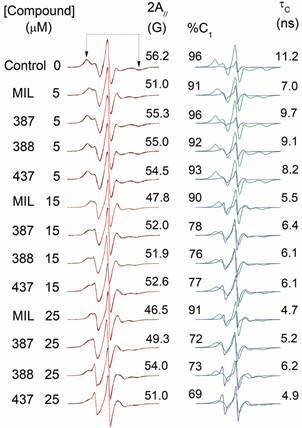
experimental (black line) and best-fit (red line) electron paramagnetic resonance (EPR) spectra of the spin label 5-DSA incorporated into the membranes of *Leishmania amazonensis* promastigotes in untreated cells (control) and cells treated with MIL and its analogs, TC387, TC388, and TC437. The EPR parameter 2A_//_ (outer hyperfine splitting), indicated by arrows, is defined as the distance in magnetic field units between the first peak and the last inverted peak of the spectrum. The estimated experimental error for the 2A_//_ parameter was 0.5 G. The total magnetic field scan range for each EPR spectrum was 100 G (X-axis), with intensity expressed in arbitrary units (Y-axis). Spectra were simulated using the NLLS program based on a two-component model. For each spectrum, the best-fitting components, C1 (green) and C2 (blue), are displayed in the right panel, along with the percentage of C1 present in the spectrum. Additionally, the calculated rotational correlation time (t_c_) values were determined according to eq. 1 and 2.

Figure 4 presents the EPR spectra of spin-label 5-DSA in the membranes of *L. amazonensis* promastigotes and the changes observed following treatment with MIL and its analogs. In *Leishmania* parasites, the MIL analogs induced plasma membrane alterations similar to those caused by MIL, albeit at concentrations approximately three times higher. The t_c_ values, derived from EPR spectral simulations, reflect the molecular dynamics of the membrane and align with observations from the 2A_//_ parameter. For samples treated with MIL analogs TC387, TC388, and TC437 at 15 μM, the EPR spectra clearly resolved two distinct spectral components. These spectra did not exhibit satisfactory convergence between theoretical and experimental data when modelled using a single-component simulation. In control samples and those treated with 5 μM of the compounds, component 1 accounted for over 90% of the spectrum. However, the fitting program likely lacks the resolution to distinguish a second component contributing less than 10%.

For samples treated with MIL, even at concentrations of 15 and 25 μM, component 1 remained above 90%. In contrast, for samples treated with 15 μM of MIL analogs, the second component represented approximately 22-28% of the spectrum. The presence of this second component suggests a population of spin labels with higher molecular dynamics (t_c_ values approximately an order of magnitude lower), indicating that MIL analogs TC387, TC388, and TC437 exhibit a non-homogeneous distribution across the parasite’s plasma membrane.

## DISCUSSION

In the present work, the leishmanicidal action of three derivatives of CNSL-alkylphosphocholine was studied in comparison with MIL. Structurally, these new compounds differ from MIL by a shortening of the linear lipophilic chain, with sixteen carbon atoms to eight oligomethylenes, and the presence of a benzene ring containing oxygenated groups (Fig. 1). These modifications likely make TC387, TC388, and TC437 less lipophilic than MIL, as supported by the lower K_M/W_ partition coefficient values observed for these compounds (Table I). However, membrane affinity may also be influenced by interactions with membrane proteins. The considerably higher K_M/W_ values for MIL suggest stronger interactions with membrane proteins than the CNSL derivatives.

Spin-label EPR spectroscopy was used to analyse MIL’s interactions with *L. amazonensis* promastigotes, employing a maleimide derivative spin label that binds covalently to sulfhydryl groups on the plasma membrane’s outer periphery.^10^ The EPR spectra showed that MIL, with a detergent-like action, significantly increased the segmental motion of the protein backbone, promoting structural changes that exposed protein regions to the solvent.^10^ ^,^ ^11^ ^,^ ^12^ These findings align with models of ionic surfactant-protein interactions,^27^ ^,^ ^28^ suggesting that MIL enters the membrane through the lipid component, acting at both the lipid-protein interface and within the hydrophobic regions of proteins, where it may form micelle-like aggregates around polypeptide chains through electrostatic and hydrophobic interactions.

Our results indicate that TC387, TC388, and TC437, due to the presence of a benzene ring attached to the alkyl chain, likely interact with the membrane in a different manner than MIL. The π-electrons of the aromatic ring may enhance their affinity for the lipid component, reducing their penetration into the protein regions and concentrating their effects at the lipid-protein interface.

EPR data using 5-DSA demonstrated that MIL and its analogs increase molecular dynamics in the membranes of *L. amazonensis* promastigotes. However, CNSL-phospholipid analogs required concentrations at least three times higher than MIL to exert equivalent effects on the parasite membrane (Fig. 4). This suggests that the TC compounds have weaker interactions with membrane proteins compared to MIL, possibly because the analogs, despite being less lipophilic, preferentially interact with the lipid component due to their aromatic rings. In samples treated with MIL analogs, two spectral components were observed in the EPR spectra (Fig. 4), indicating a second population of spin labels with greater mobility. Since the 5-DSA spin label behaves as an annular lipid that interacts with the surface of membrane proteins, it is sensitive to the molecular dynamics at the lipid-protein interface. This suggests that the CNSL-phospholipids may accumulate at the lipid-protein interface, creating more mobility or disorder in this membrane region. This interpretation is consistent with the lower c_m50_ values for MIL analogs compared to MIL (Table I), indicating that lower concentrations of MIL analogs are needed to disrupt the parasite membrane.

The high selectivity indices (SI) of TC387, TC388, and TC437 for *L. amazonensis* over erythrocytes and macrophages (SI = HC_50_/EC_50_) are notable. As shown in Table II, the HC_50_ values for MIL analogs in PBS were approximately 8 mM, while their c_w50_ (~EC_50_) values were below 40 μM (Table I), giving an SI of about 200. For J774.A1 macrophages, the CC_50_ values for MIL, TC387, TC388, and TC437 were 77 μM, 956 μM, 1633 μM, and 692 μM, respectively, while the EC_50_ values were 13 μM, 27 μM, 39 μM, and 24 μM (Fig. 2, Table III). Thus, the SI for MIL was around 6, while for TC387, TC388, and TC437, the SI values were 34, 42, and 29, respectively. The EC_50_ values were lower for the amastigote form, giving SI values much higher than those found for the MIL. It is important to note that EC_50_ values for the intracellular amastigote form can vary significantly depending on the methodology employed. For example, Scariot et al.^13^ reported IC_50_ values of 21 µM for *L. amazonensis* promastigotes and 2 µM for amastigotes internalised within J774.A1 macrophages. This selective action may be related to differences in membrane composition, such as the presence of ergosterol in *Leishmania* parasites and cholesterol in erythrocytes. Another possible explanation could involve the varying proportions of transmembrane and peripheral proteins in the cells studied. Among the CNSL derivatives, TC388 exhibited the lowest cytotoxicity in both erythrocytes and macrophages, possibly due to its less lipophilic nature compared to TC387 and TC437, as its carbomethoxy group contains more carbon and oxygen atoms (Fig. 1).

TC387, TC388, and TC437 were able to reduce *L. amazonensis* infection in murine macrophages and demonstrated superior leishmanicidal activity against intracellular amastigotes compared to MIL. To exert this effect, the compounds must penetrate two membrane barriers: the macrophage plasma membrane and the parasitophorous vacuole membrane.^29^ Given their detergent-like properties, MIL and its CNSL derivatives likely interact at the lipid-protein interfaces, facilitating passage through biological membranes. The EPR data suggest that CNSL derivatives interact more extensively with the lipid-protein interface compared to MIL, indicating less penetration into the protein component. Consequently, these derivatives may more readily permeate membranes, facilitating access to parasites internalised within macrophages. However, further studies are needed to reliably elucidate the mechanisms underlying the more pronounced activity of these compounds against amastigotes.

We hypothesise that these membrane alterations lead to electrolyte imbalances, such as Ca²⁺ influx,^14^ which could disrupt mitochondrial membrane potential and increase ROS production.^15^ ^,^ ^16^ The resulting oxidative stress likely triggers a cascade of events leading to parasite death.^19^ Promoting intracellular ROS generation is a key antimicrobial strategy.^30^ ROS, including superoxide anion (O_2_^•-^), hydrogen peroxide (H_2_O_2_), and hydroxyl radicals (^•^OH), exert broad antimicrobial effects by inducing oxidative stress.^30^ The relationship between *Leishmania* resistance to MIL and enhanced defence against ROS has been well-documented. Das et al.^31^ suggested that MIL-resistant *Leishmania* strains exhibit superior oxidative stress resilience compared to MIL-responsive strains. Furthermore, overexpression of mitochondrial iron superoxide dismutase-A (LdFeSODA) in *Leishmania donovani* has been shown to protect against MIL-induced oxidative stress, preventing programmed cell death.^32^ ^,^ ^33^

Based on EPR studies of MIL and ionic surfactant interactions with the *Leishmania* plasma membrane^12^ and the analyses of MIL analogs in this study, we propose that electrolyte leakage occurs primarily due to pore formation or defects in lipid organisation at the lipid-protein interface. The zwitterionic polar head of MIL and ionic surfactants strongly interacts with membrane proteins.^10^ ^,^ ^12^ Although the polar heads of MIL and phosphatidylcholine (PC) are structurally similar, PC is anchored to the lipid bilayer by two acyl chains, limiting its interaction with membrane proteins. In contrast, MIL and surfactants with a single acyl chain are likely to penetrate deeper into the protein component of the membrane.^24^ For MIL analogs, interactions with membrane proteins may be more localised to the lipid-protein interface. This interpretation aligns with the presence of a more mobile spectral component in the EPR spectra and the lower concentrations of these compounds required to inhibit parasite growth (smaller c_m50_, Table I).

This study investigated the antileishmanial and cytotoxic activities of the alkylphospholipid MIL and its SCNL derivatives TC387, TC388, and TC437, which feature oxygenated aromatic rings at the end of their alkyl chains. The compounds TC387, TC388, and TC437 effectively reduced *L. amazonensis* infections in murine macrophages, exhibiting significantly stronger leishmanicidal activity compared to MIL. Furthermore, these derivatives demonstrated improved selectivity indices for *L. amazonensis* promastigotes relative to erythrocytes and macrophages when compared to MIL. Using EPR spectroscopy combined with the spin-label method, we observed that MIL analogs increase the molecular dynamics of plasma membranes in *L. amazonensis* promastigotes. The lipid spin label 5-DSA, which is sensitive to mobility changes at the lipid-protein interface in biological membranes, revealed that while TC387, TC388, and TC437 induce less pronounced membrane alterations than MIL, a second spectral component was detected in the EPR spectra of 5-DSA. This suggests the presence of a more mobile population of spin labels, indicating that these analogs accumulate near the lipid-protein interface, forming regions of enhanced mobility within the membrane. The high selectivity indices of the MIL analogs TC387, TC388, and TC437 underscore their potential as therapeutic candidates. These findings strongly support further in vivo studies to evaluate the efficacy of these compounds at higher therapeutic doses, with the goal of enhancing treatment outcomes for leishmaniasis.
